# Prediction Along a Developmental Perspective in Psychiatry: How Far Might We Go?

**DOI:** 10.3389/fnsys.2021.670404

**Published:** 2021-07-06

**Authors:** Frauke Nees, Lorenz Deserno, Nathalie E. Holz, Marcel Romanos, Tobias Banaschewski

**Affiliations:** ^1^Institute of Medical Psychology and Medical Sociology, University Medical Center Schleswig Holstein, University of Kiel, Kiel, Germany; ^2^Department of Child and Adolescent Psychiatry and Psychotherapy, Central Institute of Mental Health, Medical Faculty Mannheim, Heidelberg University, Mannheim, Germany; ^3^Department of Child and Adolescent Psychiatry, Psychosomatics and Psychotherapy, University Hospital Würzburg, Würzburg, Germany; ^4^Max Planck Institute for Human Cognitive and Brain Sciences, Leipzig, Germany; ^5^Department of Psychiatry and Psychotherapy, Technische Universität Dresden, Dresden, Germany

**Keywords:** life span, prediction, modeling, developmental psychiatry, neurobiology, biomarker

## Abstract

Most mental disorders originate in childhood, and once symptoms present, a variety of psychosocial and cognitive maladjustments may arise. Although early childhood problems are generally associated with later mental health impairments and psychopathology, pluripotent transdiagnostic trajectories may manifest. Possible predictors range from behavioral and neurobiological mechanisms, genetic predispositions, environmental and social factors, and psychopathological comorbidity. They may manifest in altered neurodevelopmental trajectories and need to be validated capitalizing on large-scale multi-modal epidemiological longitudinal cohorts. Moreover, clinical and etiological variability between patients with the same disorders represents a major obstacle to develop effective treatments. Hence, in order to achieve stratification of patient samples opening the avenue of adapting and optimizing treatment for the individual, there is a need to integrate data from multi-dimensionally phenotyped clinical cohorts and cross-validate them with epidemiological cohort data. In the present review, we discuss these aspects in the context of externalizing and internalizing disorders summarizing the current state of knowledge, obstacles, and pitfalls. Although a large number of studies have already increased our understanding on neuropsychobiological mechanisms of mental disorders, it became also clear that this knowledge might only be the tip of the Eisberg and that a large proportion still remains unknown. We discuss prediction strategies and how the integration of different factors and methods may provide useful contributions to research and at the same time may inform prevention and intervention.

## Introduction

Mental disorders are the leading cause of years lived with disability (Whiteford et al., 2013), with most of them having their origin early in life during childhood and adolescence (e.g., Kessler et al., 2005, 2007; Thapar and Riglin, 2020). Despite this clear figure of disability and burden of mental disorders along with staggering economic cost (Gustavsson et al., 2011), research in developmental psychiatry and into the neurobiology of mental disorders has so far not yet led to a prolific integration of neuro-psycho-bio-social insight into the diagnostic conceptualizations, treatment, and prevention of mental disorders. We need to enhance our understanding of the underlying disease mechanisms, and to validate clinical, (neuro-)biological, and multivariate predictors of psychopathology and therapy outcome. This also includes the specific integration of individual needs and social contexts and translates treatments beyond clinical settings to individuals’ daily life.

In the present review, we will describe challenges and unmet needs in the field of developmental psychiatry commenting on (1) the current status on markers and mechanism of psychopathological pathways, (2) the lack of biological validity of diagnostic categories, and proposed approaches of symptom dimensions, (3) clinical heterogeneity, (4) the role of environmental influences and psychosocial risk and resilience factors for psychopathology, and (5) methodological advances in the field of neuroscience.

### Markers and Mechanisms of Psychopathological Pathways

A major focus in developmental psychiatry has been to understand neurobiological and psychosocial changes to identify targets for prevention and prediction strategies (Rosenberg et al., 2018). For example, for attention-deficit hyperactivity disorder (ADHD), a common mental disorder characterized by core symptoms of impulsivity, hyperactivity, and inattention (Polanczyk and Rohde, 2007), changes in executive functions, in particular, in inhibition and working memory have been reported (Faraone et al., 2015). This might be accompanied with frontostriatal and parietal hypoactivation found during inhibition (van Rooij et al., 2015) and spatial working memory in ADHD, with ADHD showing persistent difficulties with working memory operations (Martinussen et al., 2005).

Thus, obviously a wide range and diverse, rather unspecific, pattern of neuropsychobiological responses have been observed already for one type of mental disorder. It is therefore not surprising that sensitivity for only one disorder in such mechanistic links is still limited. Common mechanisms may contribute to different forms of psychopathology and their associated symptomatology. For example, in anxiety disorders, shared key features may not only range along the anxiety spectrum, but there may also be subgroups within anxiety disorders that share common mechanisms (McTeague et al., 2009, 2010, 2012; Flor and Nees, 2014). Mental health seems therefore to span along construct dimensions, reflected in neurobiological changes (Dias et al., 2015; Kotov et al., 2017). Such aspects have been proposed by the Research Domain Criteria (RDoC) Project, an initiative of the National Institute of Mental Health (Cuthbert and Insel, 2010, 2013). The RDoC project suggests to base the classification of mental disorders on ***dimensions of observable behavior and neurobiological measures*** related to these functions rather than on symptom-based descriptive categorical diagnoses. Such dimensions represent the loadings onto symptoms, where each individual receives a dimensional score. It builds a basis for parsing heterogeneity to be predicated by abnormalities in multiple distinct system- and circuit-based psychobiological changes that might be present across versus within disorders, and manifest along dimensions (Marquand et al., 2016). This is also important given that individual differences can already be observed at the subclinical stage. We have shown this for conduct problems and clinically relevant brain changes to negative affective processing in a healthy adolescent sample: regression analyses revealed a significant linear increase of left orbitofrontal cortex (OFC) activity with increasing conduct problems up to the clinical range, while in the high conduct problems group, a significant inverted u-shaped effect indicated that left OFC responses decreased again in individuals with high conduct problems (Böttinger et al., 2021).

Several studies have, however, also acknowledged a ***general, and thus one, dimension of psychopathology***, the so-called p factor, underlying multiple disorders (Caspi et al., 2014; Caspi and Moffitt, 2018). This proposition of such a single p factor for common mechanisms can be seen in line RDoC when relying on a transdiagnostic understanding, i.e., that the same mechanism underlies different diagnoses, yet it is different from RDoC when we applying the heterogeneity assumption. Using data from the longitudinal New Zealand Dunedin Multidisciplinary Health and Development Study (Caspi et al., 2020), one of the most recent child cohort studies examining children longitudinally into adulthood, it was shown that the p factor significantly improved the model fit when externalizing, internalizing, and thought disorder subfactors were integrated and allowed to inter-correlate (Caspi and Moffitt, 2018). This can be used as an evidence of common a common mechanism underlying these disorders, but, on the other hand, ignores potential individual differences within such a mechanism, for example, on subdimensions of this mechanism.

The p factor has also been shown to substantially overlap with the p factor in childhood and risk for mental disorders in adulthood (Allegrini et al., 2020), and the stability of the p factor across childhood was highly driven by genetic influences (Neumann et al., 2016). This may be further be seen as an indication of a rather static marker of psychopathology, representing an important *snap-shot*, but potentially being not such valid when describing changes and developments over time. An impact of genetic constitutions has further been unraveled through studies on the so-called polygenic risk scores (PRS) that reflects an individual’s inherited susceptibility to a disease. Higher levels of PRS for internalizing problems, for example, determined adolescents’ co-occurring internalizing/externalizing problems, indicating common genetic components for externalizing and internalizing disorders, and by lower levels of the aggression PRS through greater early childhood behavioral inhibition (Wang et al., 2020). An ADHD PRS was shown to distinguish between individuals with a persistently high level of ADHD symptoms (through to adolescence) from those individuals whose symptoms declined or remitted by adolescence (childhood-limited) (Riglin et al., 2016).

While these markers may be useful for the routine clinical practice, not all are real indicators for underlying mechanisms and useful to capture an individual disease process, having been identified through rather pragmatic pipelines. This is not an invalid procedure *per se*, but needs to be carefully considered within the respective context, i.e., whether they are used as diagnostic, prognostic, or predictive target; otherwise, their effectiveness might be strongly be limited or even misleading.

## How to Deal With the Current Knowledge on Markers and Mechanisms of Psychopathological Pathways?

Falk et al. (2013) have already raised several important questions in this respect. Among those questions, one covered brain–behavior mechanisms and interactions: “What would a ‘representative group of brains’ tell us about the generalizability of current samples and current findings regarding brain–behavior mechanisms?” This question nicely implied what we are referring to the heterogeneity *problem*, and illustrates that thinking in a one-dimensional domain, grouping individuals in comprehensive fashion, does not always also mean that we end up with more concrete and specific conclusions.

And even if we have arrived at the individual level, a following question would come up, namely, “How do individual differences in brain structure and function affect cognitive, affective, and behavioral outcomes and how do social situations and broader environmental contexts interact with these processes?” For example, several previous studies on intelligence suggested a high heritability of the intelligence quotient; however, more recent work indicates that for the whole population the heritability is not as high as previously assumed (Turkheimer et al., 2003). While the earlier studies had used samples primarily with individuals showing a high socioeconomic status (SES), more recent studies stem from more representative samples. Since the SES is a significant moderator of genetic heritability, the heritability was higher in high SES, above 70%, and in contrast with only 10% in low SES individuals (Turkheimer et al., 2003; Henrich et al., 2010), which would lead to inconsistent estimates of outcomes.

It is thus crucial to disentangle***more specifically inter-relationships among the factors of interest and map them among developmental trajectories.*** With regard to externalizing symptoms, some individuals have constantly high symptoms, while intermediate groups of individuals shift up or down slowly or rapidly. Similar patterns were observed for internalizing symptoms. For adolescence, the internalizing trajectory was also found being independent of high externalizing trajectories, and persisting externalizing problem scores were associated with decreasing internalizing scores, and early environmental risk factors and sex predicted externalizing trajectories to a larger extent than internalizing (e.g., Nivard et al., 2017). In this respect, we need to specify the relevant mechanisms of change for such shifts, and this also includes the evaluation of how the brain develops across the life span in individuals with and without mental disorders, that warrants further investigation (e.g., Baltes et al., 1977; Paus, 2010; Zelazo and Paus, 2010). Moreover, as addressed above, ***symptoms are highly heterogeneous*** and ***overlapping*** (Moffitt et al., 2007; Caspi et al., 2020). In the Dunedin Study (Caspi et al., 2020), for example, less than 15% of participating individuals diagnosed with externalizing or internalizing disorders showed a homotypic symptomatology (Caspi et al., 2020).

Strategies for understanding the etiology of mental disorders in this respect need to capitalize on data from ***longitudinal, cohort studies*** like the Mannheimer Risikokinderstudie (MARS; Laucht et al., 2000b), the Adolescent Brain Cognitive Development (ABCD) study (Karcher and Barch, 2020), the Kinder- und Jugendgesundheitssurvey (KIGGS; Mauz et al., 2019), the Saguenay Youth Study (Pausova et al., 2017), or the Imaging Genetics (IMAGEN) study (Schumann et al., 2010). Those examine the psychosocial and neurobiological etiology, prevalence, and developmental trajectories of (sub-)clinical symptoms indicative of vulnerability for future psychopathology in children, adolescents, and adults. However, so far, brain development has been investigated mainly during middle childhood (e.g., ABCD), adolescence (e.g., IMAGEN), or early adulthood (e.g., MARS). Up to date only few studies, such as IMAGEN, Generation R (Jaddoe et al., 2006), Saguenay Youth Study, or NCANDA (Rohlfing et al., 2014), pursued a longitudinal neuroimaging design. This aggravates conclusions about the cause and effect relationships, as brain changes can be both a consequence of behaviors or experiences and a causing factor.

To map and model changes over time in a non-linear fashion, study designs need to integrate more than two assessment time points, which is so far realized, for example, in IMAGEN starting during adolescence and follow-ups around every 2 years. Given the lack of neurodevelopmental studies early in life, it is still unclear whether especially early developmental curves of the brain serve as possible predictors for psychopathology, and whether and how this depends on different risk constellations and interactions between neurobiology, genes, and behavior.

It is therefore important that we obtain data very early in life along such cohort studies and longitudinal approaches starting already in birth, and apply a precise and comprehensive analysis of phenotypic abnormalities (***deep phenotyping***), integrating the individual components of the phenotype and thus being able to more specifically consider age of symptom onset and underlying psychobiosocial mechanisms (e.g., Holz et al., 2020; Thapar and Riglin, 2020). For example, about 45% of individuals who develop a mental disorder early in life reported aversive experiences during childhood (Green et al., 2010). This may depend on region-specific sensitive time windows of brain development (Callaghan and Tottenham, 2016), during which functionally neurobiological changes may occur as consequences of early negative experiences (Nelson and Gabard-Durnam, 2020). Moreover, previous etiological studies showed an earlier and more frequent manifestation at every age as well as greater stability for externalizing compared to internalizing disorders (e.g., Laucht et al., 2000a). They were related to very early psychosocial risk factors, which became apparent in early childhood and include mother–child interactions (Laucht et al., 2000a). Moreover, age of onset was discussed as one of the crucial factors for the course of symptom severity in behavioral disorders: an earlier development of symptoms is associated with stronger burden and chronification (Perra et al., 2020). Moreover, early risk factors also explain a substantial proportion of variance in behavioral and neurobiological mechanisms later in life (e.g., Holz et al., 2014, 2015) and might be important for the persistence of symptoms from child- into adulthood, as shown, for example, for ADHD or anxiety disorders (e.g., Thapar and Riglin, 2020). Thus, only the investigation of risk and resilience mechanisms already early in life enables a proper integration of this knowledge into prevention and early intervention programs.

### Environmental Influences and Neurobiological Measures: Focus on Psychosocial Risk and Resilience

Under the premise to identify neurobehavioral markers as targets for early intervention and prevention, malleability of these markers is critical. Twin studies suggest that genetic factors indeed contribute prominently to continuity in mental health problems, but that environmental influences are a major contributor to dynamic change. Further, in children younger than 5 years of age, the influence of environmental factors is even more important than genetic factors. In short, the younger the child, the more dependent and vulnerable it seems to be in relation to the surrounding environment. Importantly, children differ from adults in their unique physiological and behavioral characteristics and the potential exposure to risks in the environment (e.g., Rutter et al., 2001; Ronald, 2011; Hannigan et al., 2017).

**FIGURE 1:**
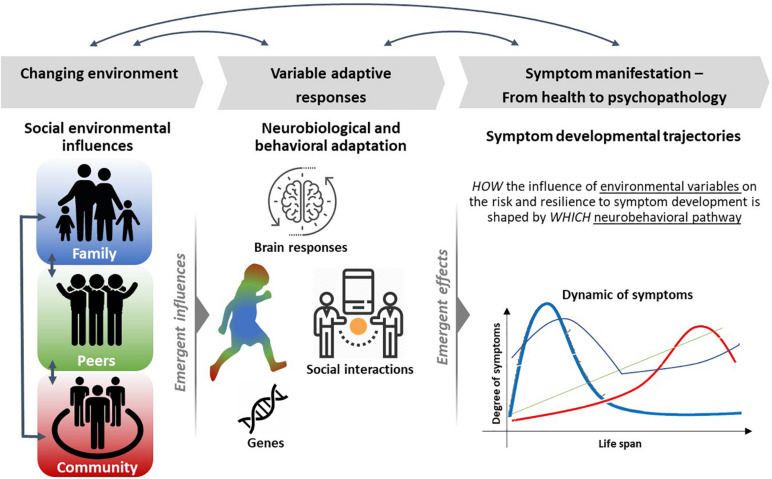
Overview on an integrative and multimodal approach to study how environmental influences dynamically shape risk and resilience on the level of neurobehavioral adaptation at different developmental stages. There are several influences through the social environment (*left*) and individuals differ in their degree to which they are exposed to environmental variation. Such variations may be reflected in neurobiopsychological and behavioral reactivity, for example, through brain–behavior relationships or gene–environmental interactions (*middle*). These variations underlie life-time-dependent changes over time and together represent trajectories of risk into and resilience for maladaptive behavior and psychopathological symptomatologies (*right*).

Along the proposed ***deep phenotyping*** approaches of psychopathology, those should therefore not only address the neurobiological, but particularly also ***environmental and psychosocial domains and their dynamic interplay*** to identify the complex etiology of mental disorders (Holz et al., 2020). We need to extent existing knowledge in neuroscience research to broader relevant populations, and the ways that macrolevel structures (e.g., social structure, neighborhood safety, school quality, and media exposure) influence neural processes (Paus, 2010), thus integrating social and neurobiological perspectives (see Figure 1).

For the social domain, it has been demonstrated that socially well-connected individuals live longer compared to those with weaker social bonds (Holt-Lunstad et al., 2010). Caregivers, including their parenting styles, are important factors for developmental processes, for example, influencing emotion regulation and emotional reactivity (Bernier et al., 2016; Holz et al., 2018). Distress of the infant can be effectively reduced by responsive caregivers decreasing the development of fear over time (Leerkes et al., 2009), also in association with brain phenotypes (Ellis et al., 2011). Interestingly, an individual’s social ties have not only beneficial health effects, but social connections also determine the way individuals perceive their surrounding environment. Loneliness and social exclusion bias perception of the social world to be more threatening or vice versa. This is related to stronger activity in the visual cortex in response to social stimuli, and thus greater attention to negative social information (Cacioppo et al., 2009). A lifestyle with infrequent and/or negative social contacts might therefore negatively affect the processing of socioemotional and reward stimuli (Etkin et al., 2006; Adolphs, 2010; Rademacher et al., 2010).

On the other hand, social support may be beneficial for well-being being an important mediator in health and disease. Specifically, receiving social support affects vmPFC brain regions that are relevant in inhibiting activity of regions, like the dorsal anterior cingulate cortex and anterior insula, associated with threat and stress processing (Eisenberger et al., 2011). Moreover, perceived social support has been shown to moderate the well-known relationships between trait anxiety and amygdala reactivity (Hyde et al., 2011). Adolescents with a negative family history and relatively severe life stress showed increased amygdala reactivity to threat (Swartz et al., 2015). Models of environmental sensitivity (Pluess, 2015) include hypotheses on sensitivity to processing sensory information, biological susceptibility to context, and differential susceptibility (Aron and Aron, 1997; Belsky et al., 2007; Boyce, 2016). It is suggested that individuals who are more sensitive show not only a higher risk for consequences of adverse environmental conditions (e.g., Monroe and Simons, 1991), but are also more responsive to positive characteristics of the environment (Pluess and Belsky, 2013). This framework may inform initiatives that aims to identify individuals who are most affected by adverse environmental influences (Meaney, 2018), and in turn, who benefit most from treatment strategies (de Villiers et al., 2018), and can also inform prevention.

With respect to associated brain changes, the amygdala and the prefrontal cortex (PFC) have been suggested to play a key role. Although the volume of the amygdala rapidly enlarges within the first years of life, structural changes process until 4 years of age in girls and 18 years in boys (Callaghan and Tottenham, 2016). Early life exposures might therefore result in alterations of brain regions like the amygdala in childhood, with an influence also of later stress specifically in boys. Aside prefrontal cortical gray matter increases are observed until adolescence, which indicates higher sensitivity to environmental influences during childhood and adolescence (Callaghan and Tottenham, 2016).

However, the research on neurobiological variability and individual differences in environmental sensitivity is scarce, and has mostly been conducted in adult and adolescent populations. Early childhood neurobiological phenotypes are still lacking. In this respect, environmental variables can act as moderators of interest and might have also contributed to the neurobiological phenotype and thus the sensitivity marker of interest. Moreover, environmental factors can also function as predictors. It also becomes clear that we would benefit from studies on brain phenotypes characterized at or shortly after birth, and thus have been only minimally influenced by environmental experiences (Nolvi et al., 2020). Connectivity between the amygdala, insula, and ventral medial PFC (vmPFC), which have been implicated in individual differences in the processing of fear and in the risk to the development of mental disorders, has also been identified as predictor of higher fear and sadness already in the newborn from 6 to 24 months of age. Specifically, amygdala–insula connectivity and amygdala–vmPFC connectivity were relevant for fear and sadness trajectories, respectively (Thomas et al., 2019), and amygdala–vmPFC connectivity at birth predicted cognitive development at 6 months of age (Nigg, 2006; Degnan and Fox, 2007; Gartstein et al., 2012).

At these very early times of life, it also becomes evident that biological influences during pregnancy are rapidly influencing developing fetal brain systems, particularly those that are altered in mental disorders. Maternal cortisol levels during pregnancy, indicating elevated levels of psychosocial stress, were significantly related to stronger connectivity between the amygdala and brain regions relevant for the processing and integration of sensory information as well as the default mode network in females and reduced amygdala connectivity to these regions in males (Graham et al., 2019). Further, in females, this connectivity mediated the association between maternal cortisol and higher internalizing symptoms (Graham et al., 2019).

### Advanced Methodological Issues to Achieve a Better Clinical Impact

Data from population-based cohort studies might help to increase the transfer into the clinical system selecting an adequate and most beneficial treatment, and particularly prevention strategy. However, the associated notion that symptom observations and reports are highly correlated within and also common across disorders (Hahn et al., 2017) does, however, not mean to completely abandon categorical approaches when parsing heterogeneity.

To pursue a promising avenue toward neuro-psycho-biological research that can have practical impact on psychiatric healthcare, we need to shift many of the traditional approaches and tools, and also need to combine approaches to build on each other. This shift becomes even more important when thinking about the still existing translation gap into clinically useful prevention and intervention strategies. A dimensional approach would result in different, individually tailored, treatment strategies depending on the occurrence of specific symptoms or additional modulators such as comorbidity of cognitive status, which could improve the treatment outcome. Methods applying categorical approaches can be added providing information on clusters or subtypes of individuals who share common features, for example, in the case where groups with ***extreme phenotypes of psychopathology*** still show alterations in multiple mechanisms.

To explore moderators of the neurobiological prediction of behavior and model clinical variance, we summarize some of the available analysis methods, which we think are reliable in this respect. This includes methodological issues like switching, e.g., from *group-level statistics* that compare clinical with non-clinical, healthy control groups, to ***approaches that characterize individual heterogeneity and developmental processes along subclinical and clinical symptom continua*** (Becht and Mills, 2020). Moreover, the use of multivariate and computational modeling and analyses to break down high-dimensional data from these large and representative samples regarding brain–behavior mechanisms is required (Falk et al., 2013; Jollans and Whelan, 2018). In this context, the propagation of a ***prediction or risk calculation*** pipelines might be a promising avenue. Information on mechanisms underlying symptom dimensions and trajectories may be implemented into risk assessment and prognosis procedures to supplement clinical decision-making.

#### Cross-Lagged Modeling

To provide support for developmental models, cross-lagged panel models (CLPMs) and autoregressive latent trajectory models with structured residuals (ALT-SR) have been used. With CLPM, as a type of discrete time structural equation modeling, panel data with two or more variables, measured at two or more time points, are analyzed. In this way, any directional effects of one variable on another variable at different time points can be estimated (Kuiper and Ryan, 2018). The ALT-SR is an extension with a crosslagged (or other) structure that is fit to the time-specific residuals from a parallel process latent growth curve model. The validity of CLPM and ALT-SR has, for example, been tested for cascades of externalizing and internalizing disorders, which have strong tendency to co-occur from childhood (Rhee et al., 2015; Martel et al., 2017). Such analyses are important for understanding the cause and nature of their co-occurrence, e.g., whether there is a directional or reciprocal causal relation and how this is mediated, and can have implications for treatment. Although analyses using CPLM and ALT-SR were consistent, the use of ALT-SR resulted in a better fit than CLPMs. Moreover, there is evidence for effects only apparent when applying the ALT-SR. This includes a negative effect of externalizing on internalizing problems in adolescence, while effects of internalizing on externalizing problems were found for both ALT-SR and CLPM (Murray et al., 2020). With typically utilized CLPMs, between- and within-person processes cannot be disaggregated, and thus their parameters reflect a difficult-to-interpret blend of the two. This disadvantage can be solved using ALT-SR (Curran et al., 2014). With ALT-SR, effects of unmeasured between-person confounds are partialed out (Berry and Willoughby, 2017).

#### Normative Modeling

**FIGURE 2:**
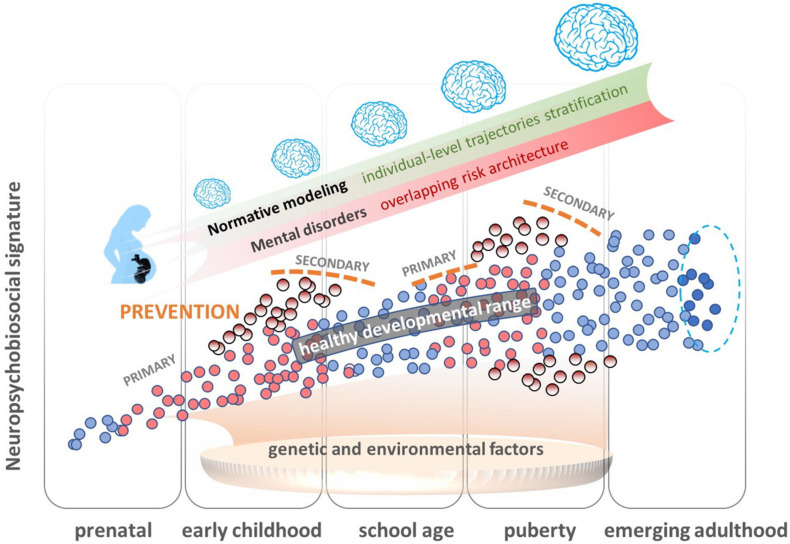
Overview of the normative modeling approach as an innovative analytical framework for parsing underlying biological heterogeneity within epidemiological cohorts without dichotomizing into cases and controls. This approach uses probabilistic regression methods, machine learning, and artificial intelligence (AI) methods to characterize variation across the population, estimate normative models of development (e.g., brain development) during critical phases of vulnerability, and detect individual differences in risk signatures in clinical cohorts. These models therefore enable predictions at an individual subject level within the population, to explore how these signatures predispose individuals to somatic and mental health outcomes, such as failure to thrive, motor, and language delay, behavioral and emotional disorders, and inferences on how deviation patterns map onto biological underpinnings informing primary and secondary prevention (early intervention) approaches. Dots represent individual neuropsychobiosocial signatures. Blue dots represent individuals who stay in the normal range throughout development, and red are those individuals who are in the normal range at a specific developmental time, but move then out of this range (pale red dots).

Normative modeling (Marquand et al., 2016, 2019; Figure 2) provides an innovative analytical framework for parsing underlying biological heterogeneity within epidemiological and clinical cohorts, providing inferences beyond the level of mean group differences. This approach uses Bayesian regression methods, such as Gaussian process regression, to characterize variation across the population as a function of clinical predictor variables, such as age, while taking predictive uncertainty into account. These models do not require deviations to overlap across individuals (e.g., in the same brain regions) and enable statistical inferences at the level of the individual participant in order to quantify deviations from the expected normative pattern from a reference cohort. In the context of psychiatry, normative modeling has been used to explain neurodevelopmental deviation patterns in clinical samples. As such, it has been applied to study developmental variability in cortical thickness (Zabihi et al., 2019) and brain asymmetry (Floris et al., 2020) in autism spectrum disorders, or gray and white matter in schizophrenia spectrum disorders (Wolfers et al., 2018). These studies have demonstrated that age-related deviations are predictive of clinical symptom scores and provided evidence that clinically relevant deviation patterns have minimal inter-subject consistency with neurobiological effects that overlap only in a few patients. In addition, spatial deviations were often different from classical case-control findings, thus corroborating the need to consider large variation across subjects at the individual level. Despite methodological challenges that may be encountered in large population-based cohorts with missing data and variance attributable to study site, normative modeling provides a promising method to estimate development (e.g., brain development) during critical phases of vulnerability and, thereby, derive individual risk and resilience signatures.

However, we also need to note that normative modeling usually requires very large sample sizes and they largely depend on the information content of the included variables. Variables representing only unprecise measures of the mechanisms of interest lower any added value at the clinical or individual level. Other machine learning and computational modeling approaches rather focus on the identification of processes and mechanisms underlying observable data, e.g., in reinforcement learning or dynamical systems. Parameters derived from these models can increase the predictive value of normative modeling as well as of other classification or clustering approaches (e.g., Brodersen et al., 2013).

#### Prediction and Risk Calculation

Aside normative modeling to perform prediction analyses, recent studies also acknowledge the development of a so-called risk calculator (Caye et al., 2020). This might be helpful for personalized medicine to predict, for example, adult ADHD from childhood characteristics, based on the representative population cohort ALSPAC-UK with 5,113 participants, followed from birth to age 17 (Caye et al., 2020). So far, the course of ADHD could not have been correctly predicted in the clinical setting based on assessments in children nor could have been prevention adequately performed for those at risk. Caye et al. (2020) therefore aimed to combine knowledge about risk factors into a multivariable risk score, similar to frameworks in the context of cardiovascular diseases, instead of using information from a single risk factor like the presence of subthreshold symptoms or of a first-degree relative diagnosed with ADHD (Brent et al., 2015; Taylor et al., 2015; Buntrock et al., 2016). They also validated their risk tool using further cohorts including the 1993 Pelotas Birth Cohort (Brazil, 3,911 participants, birth to age 18), the MTA clinical sample (United States, 476 children with ADHD and 241 controls followed for 16 years from a minimum of 8 and a maximum of 26 years old), and the E-Risk cohort (United Kingdom, 2,040 participants, birth to age 18). An add on by the knowledge and results from neurobiological studies might provide an important link to biomarker identifications.

## Outlook

A developmental perspective in psychiatry is helpful. In order to detect mental health outcomes, studying individual differences in brain development is a key aspect. However, so far most longitudinal neuroimaging studies tested effects on a group level, for example, to identify those individuals who had lower brain volume at a baseline level or also those who show accelerated brain volume at follow-up testing compared with individuals who had higher brain volumes at baseline.

To overcome this constraint, such individual differences should then also be used for prediction analyses to address heterogeneity in developmental trajectories. Future research needs to treat age not as a confound, but rather as the primary effector of interest. We need to incorporate processes of brain maturation in youth, and thus the perspective of developmental psychiatry, when determining risk and resilience factor for mental disorders. We need longitudinal large-scale studies and data, starting already early in life. Moreover, it is vital to apply a translational, transnosological, and multi-disciplinary systems approach and go beyond on brain development, which most of the available studies focus on. There is still a lack of integration of molecular, immunological, endocrinological, environmental, social, physiological, cognitive, and brain imaging readouts. Resources should include large-scale clinical (i.e., patient-based), at-risk, and epidemiological (i.e., population-based) *cohorts* that underwent comprehensive longitudinal deep phenotyping protocols, including state-of-the-art digital health technologies. Applying cutting-edge bioinformatics, we can overcome transdisciplinary research gaps and clinical service boundaries by providing multimodal predictive signatures of risk and protection. The combination with unique assembly of developmental tools, models, and human cohort readouts will allow for a deepened mechanistic understanding why children develop trajectories resulting in disease or recovery. Insight into the developmental mechanisms can then inform extensive translational intervention capacities and networks.

## Author Contributions

FN wrote the manuscript. LD, NH, MR, and TB reviewed the manuscript. All authors contributed to the article and approved the submitted version.

## Conflict of Interest

TB served in an advisory or consultancy role for ADHS digital, Infectopharm, Lundbeck, Medice, Neurim Pharmaceuticals, Oberberg GmbH, Roche, and Takeda, received conference support or speaker’s fee by Medice and Takeda, and received royalties from Hogrefe, Kohlhammer, CIP-Medien, and Oxford University Press. The present work is unrelated to the above grants and relationships. The remaining authors declare that the research was conducted in the absence of any commercial or financial relationships that could be construed as a potential conflict of interest.
